# Dimensions of genome dynamics in fungal pathogens: from fundamentals to applications

**DOI:** 10.1186/s12915-023-01786-w

**Published:** 2024-01-26

**Authors:** Daniel Croll

**Affiliations:** Laboratory of Evolutionary Genetics, Institute of Biology, University of Neuchatel, Rue Emile-Argand 11, CH-2000 Neuchâtel, Switzerland

## Abstract

**Fungi are ubiquitous** **in most ecosystems and are at the origin of serious pathogen outbreaks in humans and crops. Investigation of pathogen genomes over the past decade led to a paradigm shift of our understanding of the disease agents and provided a fertile ground for fundamental insights into drivers of genome dynamics and expression of traits.**

## Fungal pathogens as threats to human health and food production 

Fungi span a rich spectrum of lifestyles and play pivotal roles in most terrestrial ecosystems. Many species are saprotrophs, which decompose organic matter and are important in facilitating carbon cycling, or mutualists, which help plants with nutrient acquisition. Only a sliver of fungal diversity is known to be pathogenic, but these fungi are given outsized attention because of their devastating consequences for human health and agricultural production. While some fungal pathogens that affect humans are common and relatively mild, like ringworm or nail infections, others can cause frequent and serious infections, particularly in patients with a compromised immune system. Treating fungal infections is challenging given the paucity of effective drugs available and the ease with which pathogens evolve drug resistance [1]. Over the past decade, the list of the most serious and widespread human pathogens has been expanded to include *Cryptococcus neoformans*, *Aspergillus fumigatus*, and *Candida albicans* and *C. auris*. For some species, diagnostics has improved, and we have now a more accurate picture of the case load. *C. auris* is though truly a new and very serious threat increasing in frequency in hospitals across the world. From an agricultural perspective, farmers have experienced for centuries, if not millennia, the impact of fungal diseases on crops, causing steady losses or outright collapse of crops. Rust fungi epidemics are a major concern for cereal cropping, and blast diseases can be devastating for rice and wheat production. Furthermore, various soilborne pathogens cause substantial yield losses of many crops. As much as these threats to human health and food production remain significant, some powerful remedies have been discovered in recent years thanks to deep insights from fungal pathogen genomes.

## Who, when, and why? Genomic diagnostics of fungal outbreaks

If a particular crop in some part of the world starts displaying unusual disease symptoms, or a patient is suspected of carrying a fungal infection, diagnosing the identity of the pathogen is essential. A trained eye may be able to diagnose many known fungal diseases on crops by visible symptoms, and a microbiologist may be able to identify what fungus infects a patient. Yet, species identification is often only the beginning of the challenge to define adequate treatment as illustrated by the recent outbreak of wheat blast in South Asia. When wheat fields in Bangladesh started showing unexpected disease symptoms in February 2016, an international group of plant pathologists, fungal biologists, and population geneticists jumped into action. Infected leaves collected from the field were shipped to the UK for sequencing RNA directly from the samples. The international group shared immediately the sequencing data to let anyone in the world attempt to identify the nature of the disease [2]. The group discovered that the infections in Bangladesh were caused by the wheat blast fungus *Magnaporthe oryzae* typically reported only in South America. The precision in genome-based analyses of the pathogen helped establish that nearly identical genotypes of the pathogen were previously collected by collaborators in Brazil. Hence, the outbreak in Bangladesh was very likely caused by contaminated plant material imported from South America. A few years later, a nearly identical wheat blast genotype was found in a separate outbreak in Zambia. Together, this helped establish the case for a global pandemic lineage of a disease previously thought to be largely geographically restricted [3]. Knowledge of the invasive pathogen’s genome has been used to predict what antifungals are most likely effective and what wheat variety would best resist the pathogen. The fast pace to obtain and share genome sequencing data and an open, international community sharing reproducible data analyses on public websites have been an encouraging development in a scientific field that has previously preferred a more measured pace to communicate new knowledge. Studies in the UK on yellow rust outbreaks elevated such genome-informed approaches to systematically screen for signs of host adaptation and tolerance to antifungals [4]. This has enabled the scientific community to rapidly identify suitable resistant crop varieties and adjust recommendations given to farmers. Similarly, antifungals vulnerable to breakdowns in efficacy can be replaced early enough with alternative compounds or “protected” by administering these only as cocktails of multiple compounds. How broadly can such approaches be implemented at a large scale? When has resistance to antifungals emerged, and in what regions? What crop variety is most at risk of a host resistance breakdown? These are key questions in working towards the ultimate goal of being able to detect agricultural pathogen threats early and implement remedies effectively.

## The hunt for genes underlying pathogen traits in the genomics era

An explicit justification for sequencing genomes of any species is often that we want to understand the genetic basis of important traits: why do we humans suffer from certain genetic disorders? Why do turtles live long? etc. For fungal pathogens, the central question is what molecular machinery is used to infect a host? These molecular mechanisms underpin how pathogens interact with their hosts and vice versa. Human pathogens have turned out to be exceptionally good at hiding from the immune system, tolerating high body temperature, and coping with nutrient starvation, all of which contribute to pathogen “success” and difficulties in defeating them. Plant pathogens in some ways have even more sophisticated solutions to the same problems they occur during an infection. Specialized pathogens are very effective at manipulating the plant immune system to avoid detection, manipulating the host to divert nutrients to the invader, and prolonging or shortening the lifespan of plant organs depending on what confers most benefits. The agents of these pathogen trickeries are often small secreted proteins called effectors, which have little to no recognizable homology among different species. With the sequencing of plant pathogen genomes, a wealth of candidate genes encoding such effectors came to light enabling broad screens for potential functions in host infection. Despite the astonishing number of genomic resources available for nearly all economically important plant pathogens, a key question remains difficult to answer: how have pathogens gained a particular effector or reconfigured the expression of a locus? In other words, what sequence of mutations in the genome have underpinned the transition of a non-pathogen into a pathogen in the first place? Or, at a later stage, how have pathogens evolved to overcome new host resistance or to tolerate a new antifungal compound?

Sequencing of large panels of pathogen genomes of the same and similar species has been key in answering the questions around short-term pathogen adaptation. Surveying crop pathogen populations for evolutionary change has its origins in seminal applications on barley pathogens in the 1980s. Decades later, collections of hundreds and even thousands of pathogen strains from across the world have had their genomes sequenced [5]. The wealth of genomic data helped pinpoint the geographic origins of the pathogens and the time scale of invasions. Association mapping based on such pathogen genome panels revealed genes underpinning the gain of virulence and antifungal resistance [6]. Knowledge of the genetic basis of recent pathogen adaptation helps crop breeders focus on more durable resistance and directs the development towards more efficient antifungal compounds.

## Tracking the footsteps of fungal evolution one mutation (or transposon) at the time

The discovery of major loci contributing to pathogen adaptation has inadvertently revealed insights into a fundamental biological question: How adaptation of a species can be recapitulated at the genome level. For antifungal resistance, adaptive mutations are often linked to changes in the amino acid sequence of proteins targeted by the antifungal compound. In human pathogens, genes encoding targeted proteins are often duplicated to help compensate the deleterious effects of the antifungals. Genes encoding effectors in plant pathogens have undergone even more dramatic changes as the pathogen evolved to infect host plants. Drivers of this evolutionary change were often selfish elements (i.e., transposable elements) active in the pathogen genome. Transposable elements can impact a locus in which the element inserts in a multitude of ways. The simplest effect can be the disruption of a coding sequence causing loss-of-function mutants. Insertions near coding sequences can either directly affect the expression of a locus by introducing new transcription factor binding sites or indirectly affect expression by attracting epigenetic changes [7]. As a consequence, effector genes may be lost, rearranged, or silenced. Recent findings showed that the action of transposable elements on trait expression is a hallmark of much of recent adaptation across eukaryotes [8]. The compactness of fungal genomes has greatly facilitated the investigation of these rapid turnovers mediated by transposable elements and sharpened our understanding of the underlying mechanisms.

Fungal pathogens have provided a wealth of insights into how transposable elements reshape genomes, ranging from the propensity of some chromosomes to rearrange to how the epigenetic landscape shifts as a consequence of newly inserted sequences and how genomes evolved defenses to abolish the activity of selfish DNA. Long-term activity of transposable elements in pathogen genomes is also thought to be at the origin of genome compartmentalization, in other words, how chromosomes gained distinct gene-rich and gene-poor compartments. Gene-poor compartments are often highly variable among species and host key effector genes. The compartmentalization of pathogen genomes is often referred to as the “two-speed genome.” The tight association of transposable element action and effector genes, both at the level of sequence evolution and expression regulation, may have led some pathogens to develop a risky dependence. Costs of this association include increased activity of transposable elements inserting into the genome and potentially disrupting essential gene functions. This increase in TE load can impact the fitness of the organism and ultimately lead to genome expansion. The reliance on transposable elements for effector functions could be a Devil’s bargain as the short-term benefits may well turn into long-term deleterious consequences for the species [9].

## Charting the path to sustainable pathogen control

Decades of research on fungal pathogens have taught us that controlling pathogens is less like a race to win against the antagonist, but rather to race to not be outrun. Whether animal or crop pathogens, control comes most often down to either administering antifungal compounds or improving host immunity. Developing safe and effective antifungal compounds is a major priority; however, investments by public institutions or companies are at high risk because pathogens may rapidly evolve tolerance to a newly developed compound. Genomics applications can help though to identify more durable compounds by screening for early signs of resistance. In clonal pathogens, typical for human pathogens, “evolve & resequence” experiments of pathogen lines exposed to a newly developed compound can provide mechanistic insights whether resistance could easily arise in a patient receiving the drug in the future. Such information will help prioritize compounds targeting pathways that are very costly to resist. Along the same lines, diverse pathogen species could be screened early in the antifungal development process to identify naturally existing mutants that tolerate an experimental compound. Such knowledge would help steer compound development towards more durable products. Finally, pathogen genomics-informed applications such as the engineering of plant immune receptors able to recognize effectors in a made-to-order fashion [10] will help make durable crop protection take leaps in the coming decade.

## Data Availability

Not applicable.
